# Response to the article “Pharmacotherapy assessment in chronic kidney disease: validation of the pair instrument for use in Brazil”

**DOI:** 10.1590/2175-8239-JBN-2020-0129-REP

**Published:** 2020-11-23

**Authors:** Alessandra Batista Marquito, Hélady Sanders Pinheiro, Natália Maria da Silva Fernandes, Rogério Baumgratz de Paula

**Affiliations:** 1 Universidade Federal de Juiz de Fora, Faculdade de Medicina, Programa de Pós-Graduação em Saúde, Núcleo Interdisciplinar de Estudos, Pesquisas e Tratamento em Nefrologia, Juiz de Fora, MG, Brasil.; 2 Universidade Federal de Juiz de Fora, Faculdade de Medicina, Departamento de Clínica Médica, Núcleo Interdisciplinar de Estudos, Pesquisas e Tratamento em Nefrologia, Juiz de Fora, MG, Brasil.

We appreciate the careful reading of our study and the comments received, as they are relevant, demonstrate the importance of the multidisciplinary debate, and raise fundamental points in this field of research.

The article “*Pharmacotherapy assessment in chronic kidney disease: validation of the pair instrument for use in Brazil*”^1^, authored by Alessandra Batista Marquito, Hélady Sanders Pinheiro, Natália Maria da Silva Fernandes, and Rogério Baumgratz de Paula, aimed to validate the PAIR (Pharmacotherapy Assessment in Chronic Renal Disease) instrument for use in Brazilian Portuguese. Jean-François Desrochers and collaborators, from the University of Montreal, Canada^2^, originally developed this instrument.

In the present study, in line with the regulations for the validation of foreign language instruments^3^^,^ 4, we sought to remain strictly consistent with the original instrument^5^, which is why fundamental issues such as those mentioned in the letter to the editor were not included. The inclusion of new drug-related problems (DRPs) to the list of criteria in the PAIR would imply the use of a specific methodological proposal, which would cause a change to the original proposal of the PAIR creators.

In fact, the high prevalence of depression, anxiety and other disorders such as sleep disorders, leads to high consumption of psychotropic drugs that, in association with chronic kidney disease, can favor the occurrence of DRPs. However, as previously mentioned, due to the validation methodology, we had to remain consistent with the original instrument.

Thus, we hope to have clarified the questions posted in the editor’s letter, at the same time that we are grateful for the comments of our colleagues, comments that certainly open perspectives for new studies involving the evaluation of pharmacotherapy, in particular of drugs with action on the central nervous system in patients with chronic kidney disease.
